# Weather and landscape factors affect white-tailed deer neonate survival at ecologically important life stages in the Northern Great Plains

**DOI:** 10.1371/journal.pone.0195247

**Published:** 2018-04-05

**Authors:** Eric S. Michel, Jonathan A. Jenks, Kyle D. Kaskie, Robert W. Klaver, William F. Jensen

**Affiliations:** 1 Department of Natural Resource Management, South Dakota State University, Brookings, South Dakota, United States of America; 2 U.S. Geological Survey, Iowa Cooperative Fish and Wildlife Research Unit, Iowa State University, Ames, Iowa, United States of America; 3 North Dakota Game and Fish Department, Bismarck, North Dakota, United States of America; Université de Sherbrooke, CANADA

## Abstract

Offspring survival is generally more variable than adult survival and may limit population growth. Although white-tailed deer neonate survival has been intensively investigated, recent work has emphasized how specific cover types influence neonate survival at local scales (single study area). These localized investigations have often led to inconsistences within the literature. Developing specific hypotheses describing the relationships among environmental, habitat, and landscape factors influencing white-tailed deer neonate survival at regional scales may allow for detection of generalized patterns. Therefore, we developed 11 hypotheses representing the various effects of environmental (e.g., winter and spring weather), habitat (e.g., hiding and escape cover types), and landscape factors (e.g., landscape configuration regardless of specific cover type available) on white-tailed deer neonate survival up to one-month and from one- to three-months of age. At one-month, surviving fawns experienced a warmer lowest recorded June temperature and more June precipitation than those that perished. At three-months, patch connectance (percent of patches of the corresponding patch type that are connected within a predefined distance) positively influenced survival. Our results are consistent with white-tailed deer neonate ecology: increased spring temperature and precipitation are likely associated with a flush of nutritional resources available to the mother, promoting increased lactation efficiency and neonate growth early in life. In contrast, reduced spring temperature with increased precipitation place neonates at risk to hypothermia. Increased patch connectance likely reflects increased escape cover available within a neonate’s home range after they are able to flee from predators. If suitable escape cover is available on the landscape, then managers could focus efforts towards manipulating landscape configuration (patch connectance) to promote increased neonate survival while monitoring spring weather to assess potential influences on current year survival.

## Introduction

Ungulate population dynamics are influenced by multiple factors including environmental variables [1–3], density-dependent effects of forage resources [4–6], and predation [7,8]. Although adult survival rates are generally stable, juvenile survival may display more variability and thus, potentially drive population dynamics [9,10]. Therefore, understanding how factors influence juvenile survival is necessary to predict annual recruitment and its impact on population size.

Assessing how various factors such as predation and local site habitat composition influence white-tailed deer (*Odocoileus virginianus*; hereafter, deer) neonate survival has been of recent interest. Many studies have emphasized cause-specific neonate mortality [11–13] and have found coyotes (*Canis latrans*) to be the primary predator; though, predator management is often an ineffective prescription for improving population response [14, 15]. Other studies also have assessed which local habitat characteristics are associated with neonate survival across the range of white-tailed deer [12, 16–18]. Understanding which habitat characteristics influence neonate survival is important as hiding and escape cover are generally limiting in highly fragmented landscapes [19]. For example, in the Northern Great Plains, native prairie and planted grasslands have been widely converted to row crop agriculture [20], and Grovenburg et al. [21] showed that vegetation height in Conservation Reserve Program grasslands positively influenced neonate deer survival. Unfortunately, most studies assessing these relationships are site specific and inconsistencies occur [11, 22, 23]. Nevertheless, the highly fragmented landscape of the Northern Great Plains results in patches of habitat that vary in size and distribution. This landscape allows for a unique opportunity to test specific hypotheses regarding the effect of environmental (e.g., winter and spring weather), habitat (e.g., hiding and escape cover types), and landscape level factors (e.g., landscape configuration regardless of specific cover type available) on deer neonate survival and to identify general patterns at a regional scale.

Neonatal deer behaviour is heavily dependent upon age and may interact with landscape or environmental variables. For example, newborns display various predator avoidance strategies as they age, moving from a hider strategy (that incorporates fear bradycardia) early in life to a flight response from predators later in life [24, 25]. Similarly, studies show that neonate survival also varies by age [11, 26, 27], indicating that extrinsic factors likely influence neonate survival during the first weeks of life. Our objective was to assess how environmental, habitat, and landscape factors influenced deer neonate survival up to one-month (28 days) and between one- and three-months (90 days) of age using a long-term dataset collected from three states that largely comprise the Northern Great Plains (Minnesota, South Dakota, and North Dakota, USA).

We developed four general groups of hypotheses to examine how various ecological factors affect fawn survival during early life: hiding and escape cover hypotheses, weather hypotheses, a landscape configuration hypothesis, and a nutritional resource hypothesis. Our hiding and escape cover hypotheses examined whether the amount of forested area, grasslands, pasturelands, and wetlands affected neonate survival [22]. We expected that neonate survival would decrease with increasing forested cover and would increase with cover provided by grasslands, pasturelands, and wetlands. Our weather hypotheses examined the influence of winter [28–30] and spring weather [21, 31] and also examined if there was a potential lag effect of prior year weather [28] on neonate survival. Winter weather may negatively affect maternal body condition, thus influencing her ability to successfully rear offspring. Therefore, we expected that winter weather (represented by a deer winter severity index and also the independent effects of lowest winter temperature and total winter snow accumulation) would negatively affect survival. Similarly, inclement spring weather (represented with the lowest June temperature and total June precipitation) may negatively affect a neonate’s ability to thermoregulate and as a result, decrease survival. However, effects of adverse spring weather may be counteracted if proper habitat is available so we developed a thermal cover hypothesis to examine if the potentially negative effects of low June temperatures and increased total June precipitation were alleviated with increased availability of grassland, pastureland, and wetland cover.

In addition to the type and amount of hiding and escape cover that influences neonate survival, the configuration of hiding and escape cover on the landscape can also affect neonate survival [22]. We expected that neonate survival would increase with increasing patch density, connectivity among patches, and complexity of the patch shape of hiding and escape cover. Finally, row crop agriculture is prevalent in the Northern Great Plains region but is generally unavailable early in the parturition season and can negatively affect neonate survival if its presence results in a decreased amount of other hiding and escape cover [32], though we would expect that an increased amount of open water would positively affect maternal lactation efficiency [33] thus positively affecting neonate survival.

## Materials and methods

**Fig 1:**
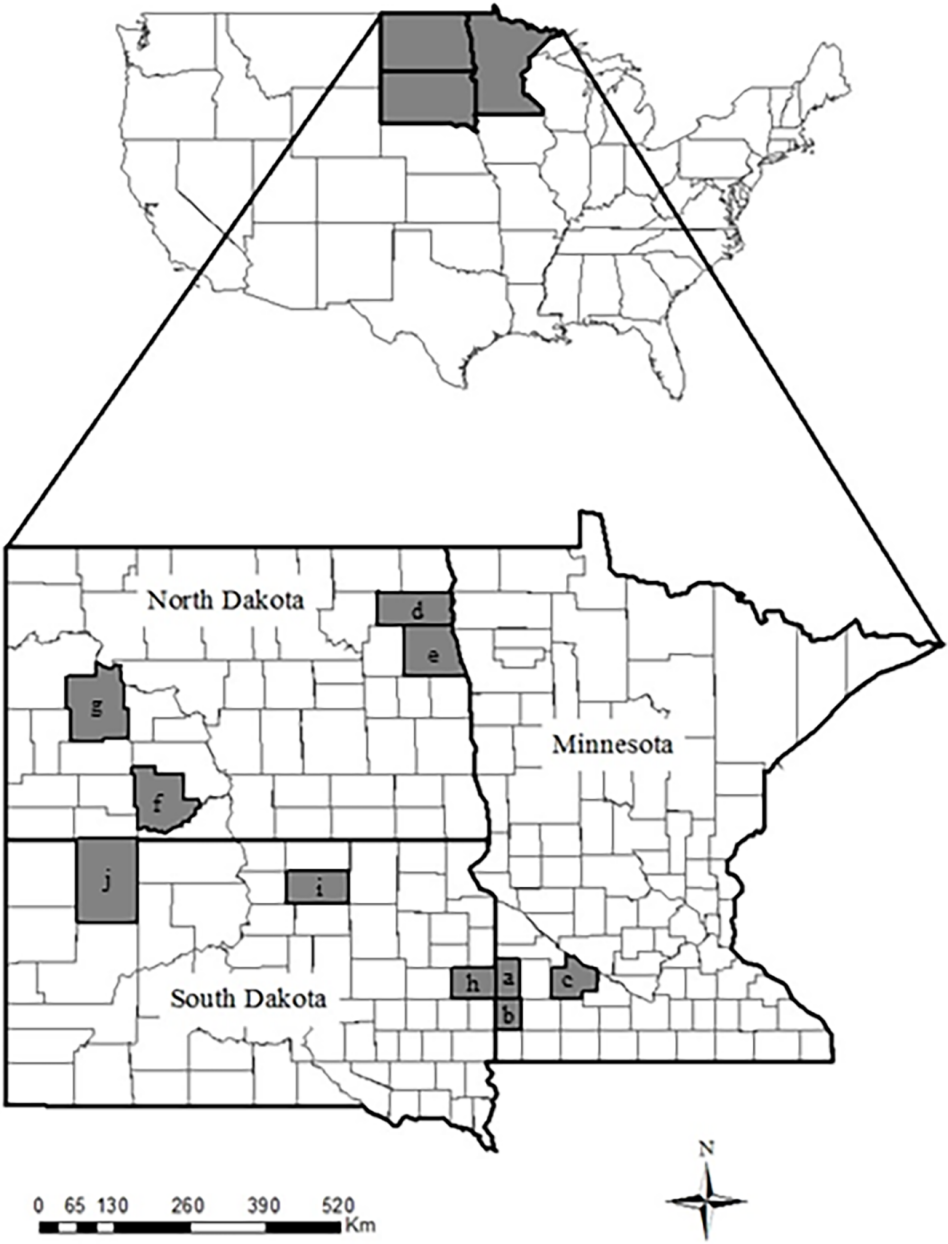
**Study sites where we captured neonate white-tailed deer located in a.) Lincoln, b.) Pipestone, and c.) Redwood counties, Minnesota; d.) Walsh, e.) Grand Forks, f.) Grant, and g.) Dunn counties North Dakota; h.) Brookings, i.) Edmunds, and j.) Perkins counties, South Dakota from 2001 to 2015**. Lincoln and Pipestone counties, Minnesota were combined to create the Lincoln-Pipestone study area and Walsh and Grand Forks counties, North Dakota were combined to create the Walsh-Grand Forks study area used for analyses.

We assessed factors that influenced neonate deer survival from 10 counties comprising 8 study sites across 3 states in the Northern Great Plains region from 2001 to 2015. We captured fawns in Walsh, Grand Forks, Grant, and Dunn counties, North Dakota; Brookings, Edmunds, and Perkins counties, South Dakota; and Lincoln, Pipestone, and Redwood counties, Minnesota (Fig 1). All counties were located within 4 Level III Ecoregions [34, 35]: Lake Agassiz Plain (Walsh and Grand Forks counties, North Dakota), Northwestern Great Plains (Grant and Dunn counties, North Dakota and Perkins County, South Dakota), Northern Glaciated Plains (Edmunds and Brookings counties, South Dakota and Lincoln County, Minnesota), and the Western Corn Belt Plains (Pipestone and Redwood counties, Minnesota). State agencies granted permission for publicly owned properties while we obtained permission from landowners to collect data from privately owned lands.

There was variation in weather variables and vegetation communities among study sites. For example, thirty-year (1981–2010) mean annual precipitation ranged from 412 mm (Grant County, North Dakota) to 729 mm (Redwood County, Minnesota), and thirty-year mean temperatures ranged from a winter low of -15.1°C (Grant County, North Dakota) to a summer high of 30.3°C (Perkins County, South Dakota [36]). Vegetation types were generally classified as Northern Wheatgrass-Needlegrass Plains, Northern Mixed Grass Prairie, and the Tallgrass Prairie [37]. As such, there was variation in the vegetation composition for each study area. Specific vegetation types are described in [11, 38, 39].

We used reproductive female postpartum behaviour as an indicator of presence of neonates [40–42], and we also used the aid of Vaginal Implant Transmitters (Advanced Telemetry Systems, Inc., Isanti, MN, USA) to assist in neonate capture [43]. We captured neonates by hand or net after locating them. We wore latex gloves and stored all radio-collars and other equipment in natural vegetation to minimize scent transfer. We fitted neonates with expandable breakaway radio-collars (Advanced Telemetry Systems, Isanti, MN, USA or Telonics Inc., Mesa, AZ). We kept handling time under five minutes when possible to reduce capture-related stress. Fawn capture methods were generally similar among study sites. For additional information regarding capture methods see [11, 22, 39].

We monitored neonates daily for the first 30 days using a truck-mounted null-peak antenna system [44], hand-held Yagi antennas (Advanced Telemetry Systems), aerial telemetry, and omnidirectional whip antennas and used these same techniques to monitor neonates 2–3 times per week thereafter. We investigated mortalities immediately after detecting a mortality signal and transported carcasses to either the North Dakota Game and Fish Department Wildlife Laboratory in Bismarck, North Dakota, USA or to the Animal Disease Research Diagnostic Laboratory at South Dakota State University, South Dakota, USA depending on study site.

### Ethics statement

We followed the American Society of Mammalogists guidelines for mammal care and use [45], and the South Dakota State University Institutional Animal Care and Use Committee approved all handling protocols (Approval numbers: 00-A038, 02-A037, 02-A043, 04-A009, 10-006E, 13-091A).

We did not calculate home ranges for each fawn as we did not have relocation data available. Instead, we quantified habitat characteristics within a 352.3-m circular buffer around a neonate’s capture location, which represented a 39-ha mean core (50%) home range for a one-month old neonate [32]. For three-month old neonates, we quantified habitat characteristics in a 669.7-m circular buffer around a neonate’s capture location, which represented a 140.9 ha (95%) summer home range [32]. We quantified habitat characteristics within a larger area for three-month old neonates to represent the increased area available to them once they began following their mother at heel. We overlaid buffered areas with the 2011 National Land Cover Database [46] and calculated habitat composition (% composition of each cover type in each buffer) using ArcGIS 10.4.1 (ESRI, Inc., Redlands CA). We then reclassified land cover data into 4 cover habitat categories (grassland/herbaceous, pastureland, wetland, and forest) and two nutritional resource categories (cultivated crop and open water).

The relative influence of the configuration of specific habitat types on neonate survival varies (e.g., [11, 22]). Our goal was to simply assess whether the amount of a specific habitat type (e.g., percent grassland/herbaceous, percent wetland) or the general landscape configuration of all habitat types found within a neonate’s home range influenced survival. Therefore, we did not test how landscape configuration of specific habitat types (e.g., number of wetland patches) may influence neonate survival; rather, we investigated how general landscape configuration, regardless of available habitat (e.g., total number of patches of all habitat types), influenced survival. We assessed how patch density, connectance, and shape index influenced survival as these variables represent the amount of escape cover available (patch density), connectivity of escape cover (connectance), and shape complexity (shape index; [47]). Examining these variables better informed us as to whether general landscape configuration or the amount of specific habitat types available more affected neonatal survival.

We obtained specific weather data from the National Oceanic and Atmospheric Administration website [48]. We used weather stations that were located closest to fawn capture locations when possible. However, full datasets were not always available from these weather stations. If unavailable, we used the next closest weather station with fully available datasets. We also calculated Deer Winter Severity Indices (DWSI, [49]) from 2000 to 2015. We awarded one point each day the mean temperature was ≤ -7°C from November to April. The index received an additional point for each day mean snow depth was ≥ 350mm during this same time period.

We developed 11 hypotheses that represent the effects of hiding and escape cover, weather, landscape configuration, and nutritional resources on neonate survival (Tables 1 and 2). We chose not to include intrinsic models (e.g., fawn body mass, fawn age, maternal body mass, maternal age) because the variation in which neonates were caught would introduce bias into the analysis [50]. Additionally, deriving specific survival rates would have been informative, but although neonates were monitored on a weekly basis, we did not have weekly encounter histories available for all neonates and thus, we only knew their ultimate fate. Therefore, we assessed factors that influenced survival up to one- and from one- to three-months using a logistic regression via the glm function in Program R (version 3.3.1; [51]). We adopted the Information–Theoretic Approach, and after analyzing each model, ranked them according the Akaike’s Information Criterion corrected for small sample size (AIC_c_) and considered models within 2 ΔAIC_c_ units as competing [52]. We derived AIC_c_ values, number of parameters, and model weights using the AIC_c_ and Weights functions in the MuMIn package in Program R [53]. We assessed correlation among explanatory variables using a Pearson’s correlation within the cor.test function in Program R and included multiple variables in a single model when |r| ≤ 0.50. We considered variables important when their 95% Confidence Intervals (95% CIs) excluded 0 [52, 54]. We also estimated $\hat{c}$ as a measure of goodness of fit of our global models [55]. We present means ± standard deviation.

**Table 1:** Names of hypotheses and variables included for each hypothesis used to explain one- and three-month neonate white-tailed deer survival in the Northern Great Plains from 2001 to 2015.

Hypothesis Name	Variables Included*	Reference(s)
Landscape	patch density (+), connectivity (+), patch shape (+)	[22]
Forested Cover	forested (-)	[22]
Grassland Cover	grassland herbaceous (+), pastureland (+), wetland (+)	[22]
Thermal Cover	lowest June temperature (-), total June precipitation (-), grassland herbaceous (+), pastureland (+), wetland (+)	[21, 22, 31]
Winter Severity Index	DWSI (-)	[28–30]
Winter Weather	total winter snow accumulation (-), lowest winter temperature (-)	[28–30]
Spring Weather	lowest June temperature (-), total June precipitation (-)	[21, 31]
Lag Effect	lowest temperature from previous June (-), lowest winter temperature (-)	[28]
Nutritional Resources	crop cover (-), open water (+)	[32]
Full	all variables included	.
Null	intercept only	.

*Predicted direction of effect included in parentheses

**Table 2:** Definition of variables used to explain one- and three-month neonate white-tailed deer survival in the Northern Great Plains from 2001 to 2015.

Variable Name	Variable Definition
Grassland Herbaceous	Percent of fawn's home range comprised of grassland herbaceous cover
Pasture Hay	Percent of fawn's home range comprised of pasture hay cover
Wetland	Percent of fawn's home range comprised of wetland cover
Forested	Percent of fawn's home range comprised of forested cover
Patch Density	Number of patches per home range
Connectance	Percent of patches connected within a predefined distance to each other within a fawns home range
Patch Shape Index	Ranges from 0 to 1; 0 represents a square patch and >0 represents deviations from the square shape
DWSI	Deer Winter Severity Index
Total Winter Snow Accumulation	Total snow accumulated from November through April
Lowest Winter Temperature	Lowest recorded temperature reported from November through April
Lowest June Temperature	Lowest recorded temperature reported in June of current parturition season
Total Precipitation in June	Total precipitation recorded in June of current parturition season
Lowest June Temperature from Previous Year	Lowest recorded temperature reported in June of previous parturition season
Percent Crop Cover	Percent of fawn's home range comprised of stand crops
Percent Open Water	Percent of fawn's home range comprised of open water

Amount of hiding and escape cover in our analyses varied because of our wide geographic range of study areas; magnitude among variables also varied (i.e., weather variables compared to cover variables). Therefore, we scaled the amount of hiding and escape cover and landscape variables by study site using the scale function in Program R with the mean centered on 0 and standard deviation of ± 1. We attempted to scale variables by county of capture; however, sample size precluded us from doing so for all counties. Therefore, we combined adjoining counties within a single state to scale variables for counties with small sample sizes. We scaled all weather variables across the dataset following the same procedure.

## Results

We captured 370 neonates from 2001 to 2015. From our one-month analysis, we censored 39 neonates, comprising 3 dropped collars, 1 lost signal, and 35 due to missing data. From our three-month analysis, we censored 105 neonates, comprising 20 dropped collars, 6 lost signals or collar malfunctions, and 66 that did not survive past one-month of age. Overall, we observed a high proportion of neonates surviving (one-month survival = 82%, one- to three-month survival = 92%). However, we observed a wide range of summer survival among populations (0.35, [39]; 0.94, [22]). Variables used within our models were not correlated (|r| ≤ 0.34). Mean distance to weather stations from neonate capture locations was 39.0 ± 24.4 km.

We observed two competing models that best described one-month survival (Table 3). Spring weather was our top model, which accounted for a moderate amount of model weight (*w*_*i*_ = 0.37). This model included the effects of total June precipitation (β = 0.313, 95% CI = 0.029–0.601, n = 331) and lowest June temperature (β = 0.334, 95% CI = 0.048–0.625, n = 331; Table 4). Our thermal cover model seemed to be competing (ΔAIC_c_ = 0.11, *w*_*i*_ = 0.35) with our top model. This model included the effects of hiding and escape cover (percent grassland/herbaceous, wetland, and pastureland cover), total June precipitation, and the lowest recorded June temperature. However, 95% CIs overlapped 0 for cover variables but excluded 0 for total June precipitation (β = 0.318, 95% CI = 0.031–0.608, n = 331) and lowest June temperature (β = 0.302, 95% CI = 0.013–0.596, n = 331; Table 4); therefore, we concluded that hiding and escape cover variables were uninformative [54] and only interpret our spring weather model. McFadden’s R^2^ for our spring weather model was 0.02 and $\hat{c}$ was 0.92 indicating overdispersion of the data did not occur [55]. All other models were ≥3.32 ΔAIC_c_ units away from our top model.

**Table 3:** Candidate set of ecological models describing one-month neonate white-tailed deer survival in the Northern Great Plains from 2001 to 2015. Models within 2 ΔAIC_c_ are competing, *w*_*i*_ indicates model weight, and *K* indicates number of parameters calculated within a model.

Model	AICc	ΔAICc	*w*_*i*_	*K*
Spring Weather	320.13	0.00	0.37	3
Thermal Cover	320.24	0.11	0.35	6
Grassland Cover	323.36	3.22	0.07	4
Null	324.21	4.08	0.05	1
Winter Weather	324.28	4.15	0.05	3
Full	324.88	4.75	0.03	15
Lag Effect	325.29	5.16	0.03	3
Forested Cover	325.73	5.60	0.02	2
Winter Severity Index	325.74	5.61	0.02	2
Landscape	327.48	7.34	0.01	4
Nutritional Resources	327.56	7.42	0.01	3

**Table 4:** Beta estimates and 95% Confidence Intervals (95% CIs) of variables used to estimate one-month neonate white-tailed deer survival in the Northern Great Plains from 2001 to 2015. 95% CIs excluding 0 indicate variable influenced survival.

Model	Variable	β	95% CI
Spring Weather	Total June Precipitation	0.313	0.029–0.601
	Lowest June Temperature	0.334	0.048–0.625
Thermal Cover	Total June Precipitation	0.318	0.031–0.608
	Lowest June Temperature	0.302	0.013–0.596
	Percent Grassland Herbaceous Cover	-0.232	-0.507–0.046
	Percent Wetland Cover	0.199	-0.106–0.557
	Percent Pastureland Cover	0.153	-0.140–0.467

**Fig 2:**
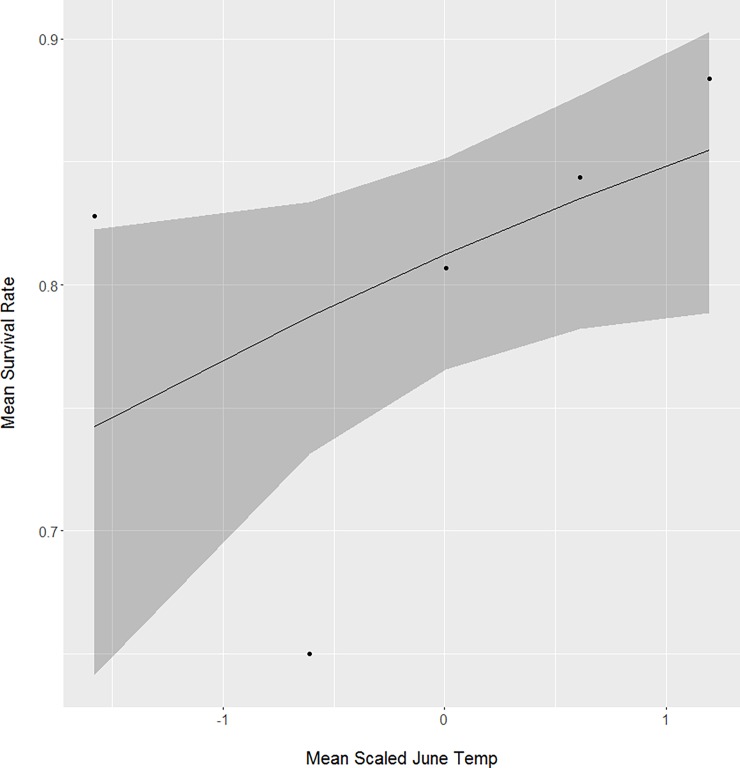
The positive relationship between mean lowest recorded June temperature and one-month survival for neonate white-tailed deer captured from Minnesota, South Dakota, and North Dakota, USA from 2001 to 2015.

**Fig 3:**
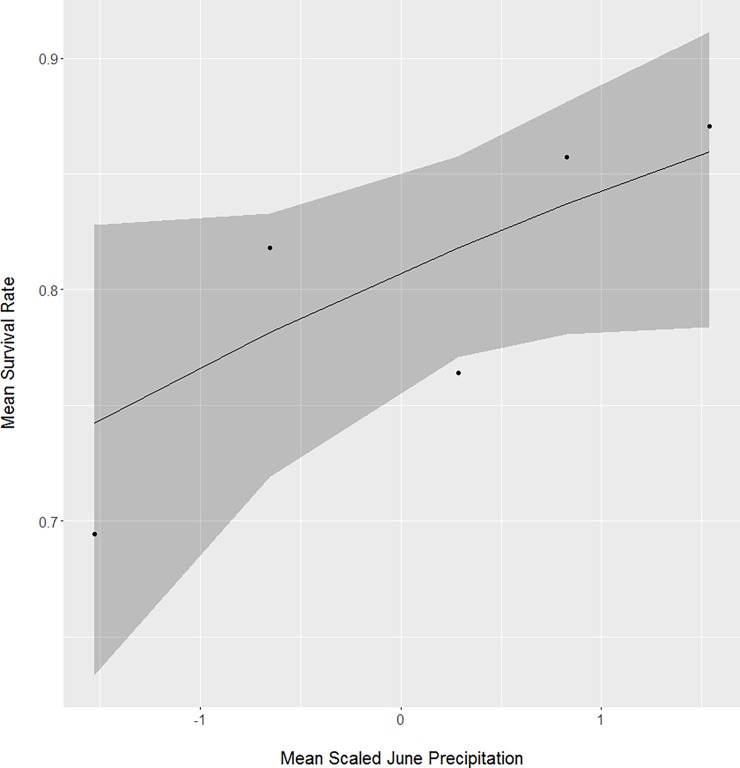
The positive relationship between mean total recorded June precipitation and one-month survival for neonate white-tailed deer captured from Minnesota, South Dakota, and North Dakota, USA from 2001 to 2015.

Both lowest recorded temperature in June and total June precipitation positively influenced one-month neonate survival. The mean lowest recorded temperature in June for surviving neonates ($\bar{x}$ = 4.7 ± 1.8°C, n = 268; Fig 2) was about 11% warmer than for neonates that perished ($\bar{x}$ = 4.2 ± 1.7°C, n = 63). For mean total June precipitation, surviving neonates ($\bar{x}$ = 111 ± 27 mm, n = 268; Fig 3) experienced about 7% more rainfall than neonates that perished ($\bar{x}$ = 104 ± 28 mm, n = 63).

**Fig 4:**
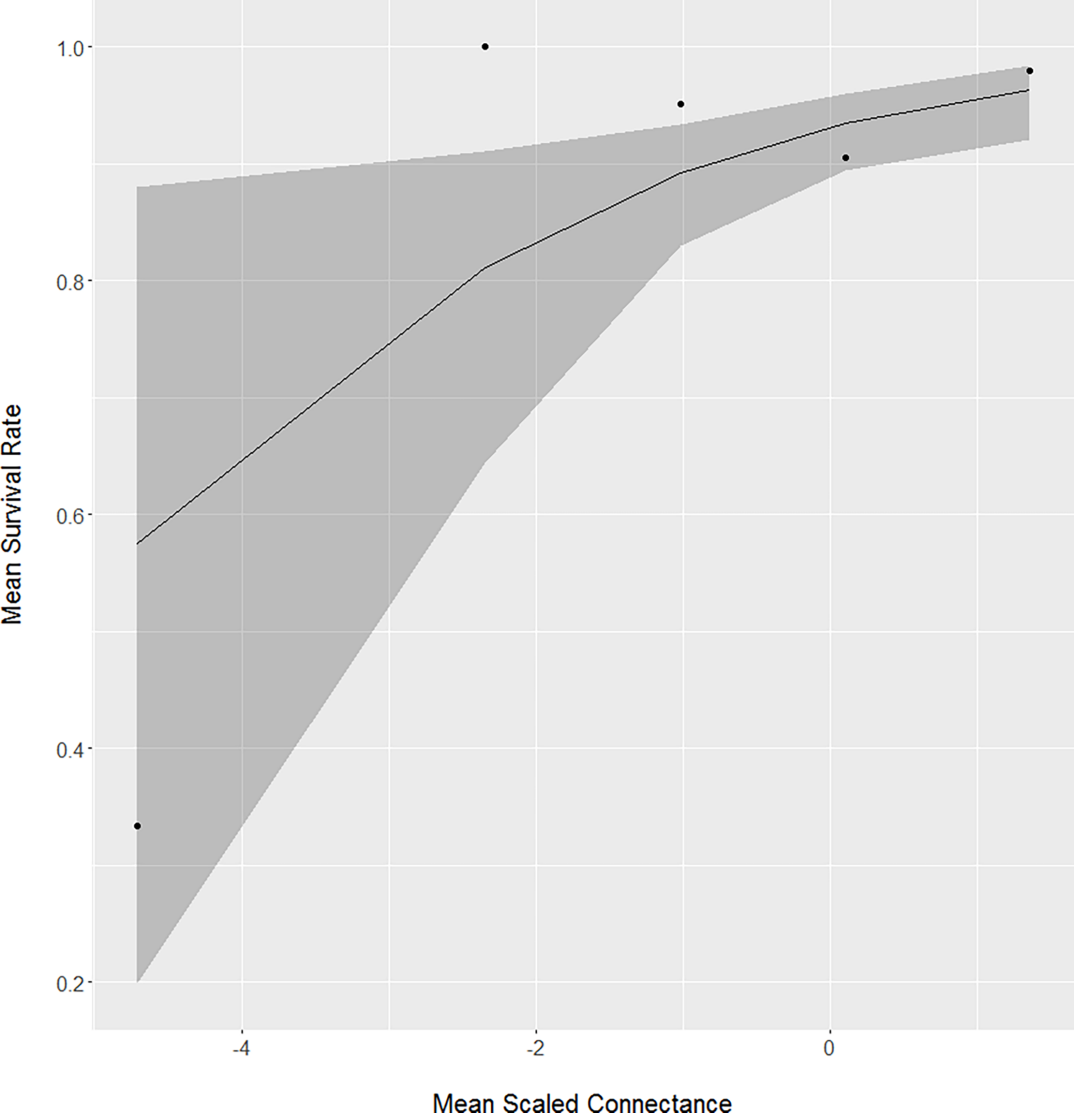
The positive relationship between mean connectance and survival between one- and three-months for neonate white-tailed deer captured from Minnesota, South Dakota, and North Dakota, USA from 2001 to 2015.

We observed one top model describing survival from one- to three-months (Table 5). Our top model was our landscape model (*w*_i_ = 0.50), which included the influence of patch density, connectance, and shape index, with 95% CIs excluding 0 for connectance (β = 0.450, 95% CI = 0.079–0.843, n = 265; Table 6). McFadden’s R^2^ for our spring weather model was 0.06 and $\hat{c}$ for our global model was 0.49 indicating overdispersion of the data did not occur [55]. All other models were ≥ 2.44 ΔAIC_c_ units from the top model. Surviving neonates ($\bar{x}$ = 77.0 ± 13.3%, n = 245; Fig 4) were exposed to about 13% more connectance among patches compared to neonates that perished ($\bar{x}$ = 68.1 ± 24.3%, n = 20).

**Table 5:** Candidate set of ecological models describing survival between one- and three-months for neonate white-tailed deer in the Northern Great Plains from 2001 to 2015. *w*_*i*_ indicates model weight, and *K* indicates number of parameters calculated within a model.

Model	AIC_c_	ΔAIC_c_	*w*_*i*_	*K*
Landscape	141.39	0.00	0.50	4
Null	143.83	2.44	0.15	1
Nutritional Resources	144.92	3.53	0.09	3
Winter Severity Index	145.73	4.35	0.06	2
Forested Cover	145.85	4.46	0.05	2
Grassland Cover	145.95	4.57	0.05	4
Lag Effect	146.83	5.45	0.03	3
Winter Weather	147.07	5.68	0.03	3
Spring Weather	147.26	5.88	0.03	3
Thermal Cover	149.53	8.14	0.01	6
Full	158.46	17.07	0.00	16

**Table 6:** Beta estimates and 95% Confidence Intervals (95% CIs) of variables used to estimate survival between one- and three-months for neonate white-tailed deer in the Northern Great Plains from 2001 to 2015. 95% CIs excluding 0 indicate variable influenced survival.

Model	Variable	β	95% CI
Landscape	Connectance	0.450	0.079–0.843
	Patch Density	0.320	-0.154–0.813
	Patch Shape	0.242	-0.211–0.716

## Discussion

We acknowledge that our low R^2^ values indicate low predictive power for our top models, though R^2^ values associated with logistic models should be interpreted with caution [56]. The low predictive power of our top models may be related to the relatively high proportion of fawns that survived to one-month (82%) and from one- to three-months of age (92%), which likely reduced the amount of variation that could be explained in our models. Conversely, there may have been little variability associated with our explanatory variables and/or our explanatory variables may have served as a proxy for another unmeasured variable. Therefore, although we obtained a large sample size (one-month survival = 331 neonates and three-month survival = 265 neonates) with high spatial (10 counties spanning three states) and temporal (15-years) replication, interpretation and use of our results for management purposes should be done so with caution.

Our results support our spring weather hypothesis and suggest that two spring weather variables most influence one-month neonate survival, though the direction of relationships differed from what we expected. We predicted that lowest June temperature and total June precipitation would negatively influence one-month survival due to neonates being unable to thermoregulate at a young age [21, 31]. However, we found positive relationships between lowest June temperature and total June precipitation and one-month survival, indicating that survival increased with increasing temperature and precipitation during June. This positive effect indicates that neonates experiencing warmer and wetter springs may be better able to thermoregulate compared those experiencing cooler and dryer springs. Alternatively, neonates may be more affected by maternal care related to forage quality and quantity during spring months than the inability to thermoregulate. For example, increased temperatures and precipitation were related to increased plant quantity and quality [57, 58], which may indirectly influence neonate survival through improved maternal lactation efficiency [59, 60]. Our results also support previous literature discussing the importance of available forage during maternal lactation [61–63] and other research reporting increased neonate survival following increased rain events [4] and warmer temperatures [64] during spring. Spring weather may therefore be an important factor for understanding neonatal deer survival, though small annual changes (≤ 11% difference in June temperature and precipitation between surviving neonates and those that perished) likely do not greatly impact overall survival as 82% of neonates in our study survived to one-month.

Our results describing neonate survival between one- and three-months supported our landscape hypothesis with patch connectance being the only important variable in the model. Our findings support Grovenburg et al. [65] who found that the probability of deer neonates eluding predators decreased with increased distance to grassland and wetland patches (i.e., areas with less connectance). Landscape metrics also have been found to influence neonate survival in other studies [22, 27, 66]. Grovenburg et al. [22] found that patch density of grassland and wetland cover types positively influenced neonate survival, while Rohm et al. [27] reported that surviving neonates inhabited home ranges that contained few large and irregular shaped forest patches. Gulsby et al. [66] also reported that coyote depredation on neonate white-tailed deer decreased with increasing amount of edge found within neonate’s home ranges. Increased escape cover and its proximity to a neonate increases probability of survival, but again, the magnitude of its impact is likely not great considering the relatively high proportion (92%) of fawns surviving from one- to three-months of age.

Lack of hiding and escape cover may potentially influence population dynamics for generalist species such as deer [19]. Our results show that general landscape configuration (connectance) and not specific cover type (percent grassland/herbaceous, wetland, and pastureland) influenced neonate survival; however, reported effects of hiding and escape cover on neonate survival is inconsistent. For example, in northcentral South Dakota, USA, the percent of Conservation Reserve Program grasslands and wetlands found in a neonate’s home range positively influenced survival, while percent forested cover negatively influenced neonate survival [32]. Also, percent forested cover positively influenced neonate survival in southern Illinois [27]. However, percent wetland, cropland, grassland, and forested cover found in a neonate’s home range did not influence survival in South Dakota, USA and Minnesota, USA [11], nor did percent herbaceous cover influence neonate survival in Pennsylvania, USA [17]. Our cover habitat variables described the amount of hiding cover available to a neonate, not use, and therefore, may only be coarsely related to neonate survival. Similarly, microhabitat characteristics do not seem to better explain neonate survival, as Chitwood et al. [13] failed to detect any influence of vegetation associated with bed site characteristics on fawn survival in North Carolina, USA. Therefore, general landscape configuration in addition to the type and amount of hiding and escape cover present could be assessed when studying factors that affect neonate survival.

Our results did not support our winter weather hypotheses for one- and between one- and three-month survival and were outperformed by the null model in both candidate sets. Winter weather negatively influenced parturition date, birth mass, and litter size for Soay sheep (*Ovis aries*; [67]), fall fawn recruitment of mule deer (*O*. *hemionus*; [28]), and negatively influenced overwinter survival during an offspring’s first year of life (elk, *Cervus elaphus*, [29]; mule deer, [30]). However, winter weather did not influence calf body mass (reindeer, *Rangifer tarandus*; [68]) or neonate survival (white-tailed deer, [69]; elk, [7]). These discrepancies may be related to an individual species breeding strategy. For example, although white-tailed deer display tendencies of both a capital and income breeder (discussed in [70]), the greatest portion of fetal development occurs in the third trimester, which generally coincides with spring green-up [71, 72]. This delayed developmental strategy allows female white-tailed deer to avoid negative effects of severe winters on their offspring’s development while in utero.

Our goal was not to assess cause-specific mortality of white-tailed deer neonates; yet, it is important to note that coyotes are generally the leading cause of neonate mortality in this region [11, 16, 22]. Our finding that increased patch connectance is positively related to neonate survival between one- and three-months of age indicates that, within a neonate’s home range, manipulating habitat already found on the landscape may have a negative effect on a coyote’s search efficiency. However, increasing the amount of hiding and escape cover available through active habitat management to promote increased quantity and quality of individual bed sites did not improve fawn survival in the southeastern United States [13, 14] where fawn survival is substantially lower (as low as 14%; [13]) than what we report for our study. Nevertheless, active habitat management has other benefits such as increasing forage quantity [73] which, in turn, may positively influence lactation efficiency [53, 54]. Future research in the highly fragmented landscape in the Northern Great Plains could focus on addressing how manipulating the landscape may disrupt a coyote’s search pattern, which may decrease their effectiveness as a predator of neonatal deer.

## Conclusions

White-tailed deer are a highly managed species in North America with a current emphasis being placed on assessing factors influencing neonate recruitment [10, 13, 74]. Our results can be used by managers in the Northern Great Plains to improve neonate hiding cover, though managers must be realistic with their expectations regarding the magnitude of increased neonate survival. Regardless, if suitable cover is available on the landscape, then managers could focus efforts on manipulating landscape configuration rather than promoting specific cover types. Managers may accomplish this by increasing the connectance (percent of patches of a corresponding patch type) within 140.9 ha sections representing a neonate’s 95% core summer home range. Doing so will increase the escape cover available to a neonate at a large scale. Additionally, managers could monitor spring weather variables such as lowest recorded June temperature and amount of June precipitation so those parameters can be incorporated into population models allowing for more refined population estimates.

## Supporting information

S1 File Data used to assess factors that influenced white-tailed deer neonate survival up to one-month of age. (XLSX)

S2 File Data used to assess factors that influenced white-tailed deer neonate survival between one- and three-months of age. (XLSX)
